# A scaling normalization method for differential expression analysis of RNA-seq data

**DOI:** 10.1186/gb-2010-11-3-r25

**Published:** 2010-03-02

**Authors:** Mark D Robinson, Alicia Oshlack

**Affiliations:** 1 Bioinformatics Division, Walter and Eliza Hall Institute, 1G Royal Parade, Parkville 3052, Australia; 2 Epigenetics Laboratory, Cancer Program, Garvan Institute of Medical Research, 384 Victoria Street, Darlinghurst, NSW 2010, Australia

## Abstract

The fine detail provided by sequencing-based transcriptome surveys suggests that RNA-seq is likely to become the platform of choice for interrogating steady state RNA. In order to discover biologically important changes in expression, we show that normalization continues to be an essential step in the analysis. We outline a simple and effective method for performing normalization and show dramatically improved results for inferring differential expression in simulated and publicly available data sets.

## Abstract 2

A novel and empirical method for normalization of RNA-seq data is presented

## Background

The transcriptional architecture is a complex and dynamic aspect of a cell's function. Next generation sequencing of steady state RNA (RNA-seq) gives unprecedented detail about the RNA landscape within a cell. Not only can expression levels of genes be interrogated without specific prior knowledge, but comparisons of expression levels between genes within a sample can be made. It has also been demonstrated that splicing variants [1,2] and single nucleotide polymorphisms [3] can be detected through sequencing the transcriptome, opening up the opportunity to interrogate allele-specific expression and RNA editing.

An important aspect of dealing with the vast amounts of data generated from short read sequencing is the processing methods used to extract and interpret the information. Experience with microarray data has repeatedly shown that normalization is a critical component of the processing pipeline, allowing accurate estimation and detection of differential expression (DE) [4]. The aim of normalization is to remove systematic technical effects that occur in the data to ensure that technical bias has minimal impact on the results. However, the procedure for generating RNA-seq data is fundamentally different from that for microarray data, so the normalization methods used are not directly applicable. It has been suggested that 'One particularly powerful advantage of RNA-seq is that it can capture transcriptome dynamics across different tissues or conditions without sophisticated normalization of data sets' [5]. We demonstrate here that the reality of RNA-seq data analysis is not this simple; normalization is often still an important consideration.

Current RNA-seq analysis methods typically standardize data between samples by scaling the number of reads in a given lane or library to a common value across all sequenced libraries in the experiment. For example, several authors have modeled the observed counts for a gene with a mean that includes a factor for the total number of reads [6-8]. These approaches can differ in the distributional assumptions made for inferring differences, but the consensus is to use the total number of reads in the model. Similarly, for LONG-SAGE-seq data, 't Hoen *et al*. [9] use the square root of scaled counts or the beta-binomial model of Vencio *et al*. [10], both of which use the total number of observed tags. For normalization, Mortazavi *et al*. [11] adjust their counts to reads per kilobase per million mapped (RPKM), suggesting it 'facilitates transparent comparison of transcript levels both within and between samples.' By contrast, Cloonan *et al*. [12] log-transform the gene length-normalized count data and apply standard microarray analysis techniques (quantile normalization and moderated t-statistics). Sultan *et al*. [2] normalize read counts by the 'virtual length' of the gene, the number of unique 27-mers in exonic sequence, as well as by the total number of reads. Recently, Balwierz *et al*. [13] illustrated that deepCAGE (deep sequencing cap analysis of gene expression) data follow an approximate power law distribution and proposed a normalization strategy that equates the read count distributions across samples.

Scaling to library size as a form of normalization makes intuitive sense, given it is expected that sequencing a sample to half the depth will give, on average, half the number of reads mapping to each gene. We believe this is appropriate for normalizing between replicate samples of an RNA population. However, library size scaling is too simple for many biological applications. The number of tags expected to map to a gene is not only dependent on the expression level and length of the gene, but also the composition of the RNA population that is being sampled. Thus, if a large number of genes are unique to, or highly expressed in, one experimental condition, the sequencing 'real estate' available for the remaining genes in that sample is decreased. If not adjusted for, this sampling artifact can force the DE analysis to be skewed towards one experimental condition. Current analysis methods [6,11] have not accounted for this proportionality property of the data explicitly, potentially giving rise to higher false positive rates and lower power to detect true differences.

The fundamental issue here is the appropriate metric of expression to compare across samples. The standard procedure is to compute the proportion of each gene's reads relative to the total number of reads and compare that across all samples, either by transforming the original data or by introducing a constant into a statistical model. However, since different experimental conditions (for example, tissues) express diverse RNA repertoires, we cannot always expect the proportions to be directly comparable. Furthermore, we argue that in the discovery of biologically meaningful changes in expression, it should be considered undesirable to have under- or oversampling effects (discussed further below) guiding the DE calls. The normalization method presented below uses the raw data to estimate appropriate scaling factors that can be used in downstream statistical analysis procedures, thus accounting for the sampling properties of RNA-seq data.

## Results and discussion

### A hypothetical scenario

Estimated normalization factors should ensure that a gene with the same expression level in two samples is not detected as DE. To further highlight the need for more sophisticated normalization procedures in RNA-seq data, consider a simple thought experiment. Imagine we have a sequencing experiment comparing two RNA populations, A and B. In this hypothetical scenario, suppose every gene that is expressed in B is expressed in A with the same number of transcripts. However, assume that sample A also contains a set of genes equal in number and expression that are not expressed in B. Thus, sample A has twice as many total expressed genes as sample B, that is, its RNA production is twice the size of sample B. Suppose that each sample is then sequenced to the same depth. Without any additional adjustment, a gene expressed in both samples will have, on average, half the number of reads from sample A, since the reads are spread over twice as many genes. Therefore, the correct normalization would adjust sample A by a factor of 2.

The hypothetical example above highlights the notion that the proportion of reads attributed to a given gene in a library depends on the expression properties of the whole sample rather than just the expression level of that gene. Obviously, the above example is artificial. However, there are biological and even technical situations where such a normalization is required. For example, if an RNA sample is contaminated, the reads that represent the contamination will take away reads from the true sample, thus dropping the number of reads of interest and offsetting the proportion for every gene. However, as we demonstrate, true biological differences in RNA composition between samples will be the main reason for normalization.

### Sampling framework

A more formal explanation for the requirement of normalization uses the following framework. Define *Y*_*gk *_as the observed count for gene *g *in library *k *summarized from the raw reads, *μ*_*gk *_as the true and unknown expression level (number of transcripts), *L*_*g *_as the length of gene *g *and *N*_*k *_as total number of reads for library *k*. We can model the expected value of *Y*_*gk *_as:

*S*_*k *_represents the total RNA output of a sample. The problem underlying the analysis of RNA-seq data is that while *N*_*k *_is known, *S*_*k *_is unknown and can vary drastically from sample to sample, depending on the RNA composition. As mentioned above, if a population has a larger total RNA output, then RNA-seq experiments will under-sample many genes, relative to another sample.

At this stage, we leave the variance in the above model for *Y*_*gk *_unspecified. Depending on the experimental situation, Poisson seems appropriate for technical replicates [6,7] and Negative Binomial may be appropriate for the additional variation observed from biological replicates [14]. It is also worth noting that, in practice, the *L*_*g *_is generally absorbed into the *μ*_*gk *_parameter and does not get used in the inference procedure. However, it has been well established that gene length biases are prominent in the analysis of gene expression [15].

### The trimmed mean of M-values normalization method

The total RNA production, *S*_*k*_, cannot be estimated directly, since we do not know the expression levels and true lengths of every gene. However, the relative RNA production of two samples, *f_k _= S_k_/S_k'_*, essentially a global fold change, can more easily be determined. We propose an empirical strategy that equates the overall expression levels of genes between samples under the assumption that the majority of them are not DE. One simple yet robust way to estimate the ratio of RNA production uses a weighted trimmed mean of the log expression ratios (trimmed mean of M values (TMM)). For sequencing data, we define the gene-wise log-fold-changes as:

and absolute expression levels:

To robustly summarize the observed M values, we trim both the M values and the A values before taking the weighted average. Precision (inverse of the variance) weights are used to account for the fact that log fold changes (effectively, a log relative risk) from genes with larger read counts have lower variance on the logarithm scale. See Materials and methods for further details.

For a two-sample comparison, only one relative scaling factor (*f*_*k*_) is required. It can be used to adjust both library sizes (divide the reference by  and multiply non-reference by ) in the statistical analysis (for example, Fisher's exact test; see Materials and methods for more details).

Normalization factors across several samples can be calculated by selecting one sample as a reference and calculating the TMM factor for each non-reference sample. Similar to two-sample comparisons, the TMM normalization factors can be built into the statistical model used to test for DE. For example, a Poisson model would modify the observed library size to an effective library size, which adjusts the modeled mean (for example, using an additional offset in a generalized linear model; see Materials and methods for further details).

### A liver versus kidney data set

**Figure 1:**
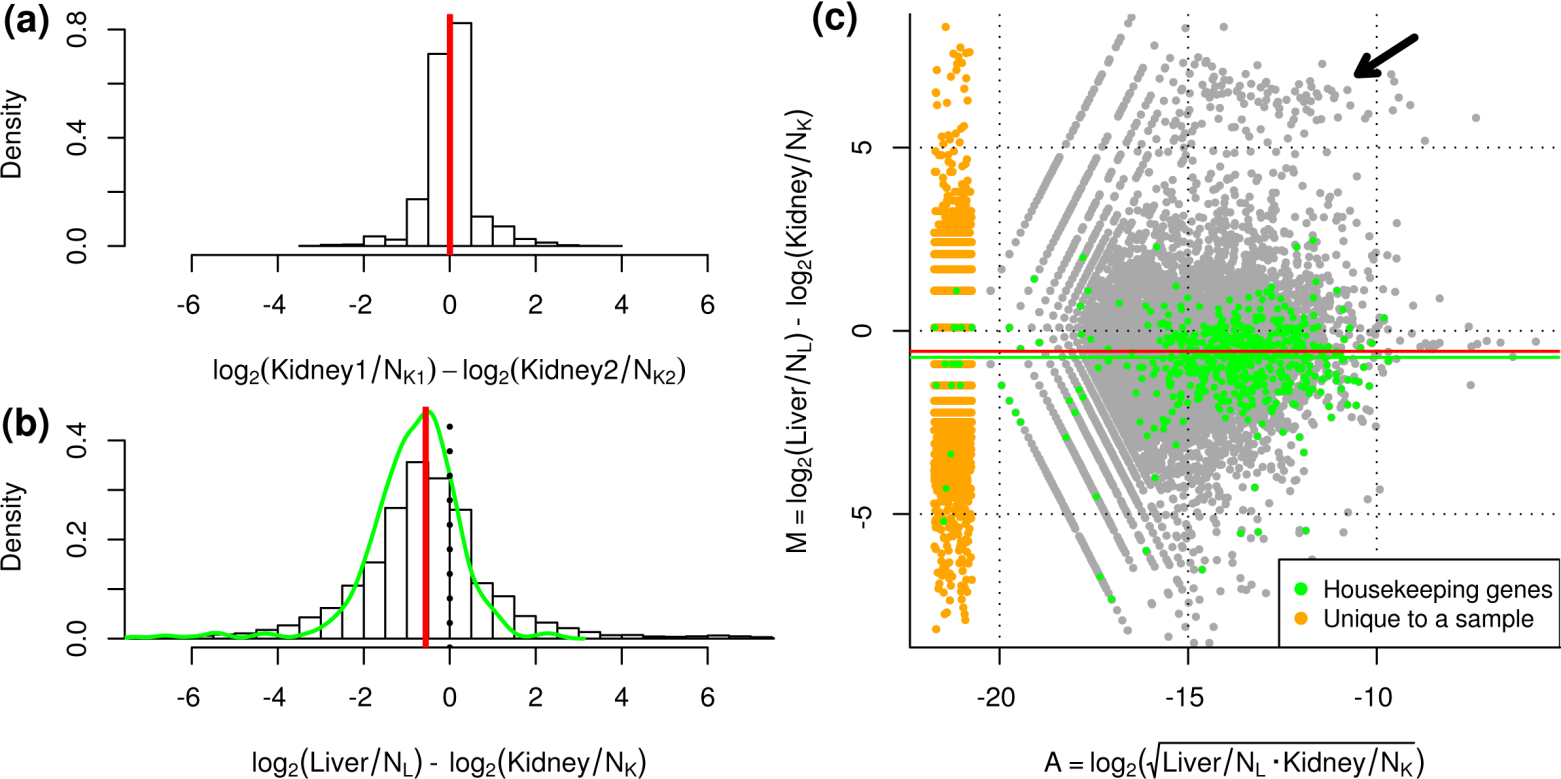
**Normalization is required for RNA-seq data**. Data from [6] comparing log ratios of **(a) **technical replicates and **(b) **liver versus kidney expression levels, after adjusting for the total number of reads in each sample. The green line shows the smoothed distribution of log-fold-changes of the housekeeping genes. **(c) **An M versus A plot comparing liver and kidney shows a clear offset from zero. Green points indicate 545 housekeeping genes, while the green line signifies the median log-ratio of the housekeeping genes. The red line shows the estimated TMM normalization factor. The smear of orange points highlights the genes that were observed in only one of the liver or kidney tissues. The black arrow highlights the set of prominent genes that are largely attributable for the overall bias in log-fold-changes.

We applied our method to a publicly available transcriptional profiling data set comparing several technical replicates of a liver and kidney RNA source [6]. Figure 1a shows the distribution of M values between two technical replicates of the kidney sample after the standard normalization procedure of accounting for the total number of reads. The distribution of M values for these technical replicates is concentrated around zero. However, Figure 1b shows that log ratios between a liver and kidney sample are significantly offset towards higher expression in kidney, even after accounting for the total number of reads. Also highlighted (green line) is the distribution of observed M values for a set of housekeeping genes, showing a significant shift away from zero. If scaling to the total number of reads appropriately normalized RNA-seq data, then such a shift in the log-fold-changes is not expected. The explanation for this bias is straightforward. The M versus A plot in Figure 1c illustrates that there exists a prominent set of genes with higher expression in liver (black arrow). As a result, the distribution of M values (liver to kidney) is skewed in the negative direction. Since a large amount of sequencing is dedicated to these liver-specific genes, there is less sequencing available for the remaining genes, thus proportionally distorting the M values (and therefore, the DE calls) towards being kidney-specific.

The application of TMM normalization to this pair of samples results in a normalization factor of 0.68 (-0.56 on log2 scale; shown by the red line in Figure 1b, c), reflecting the under-sampling of the majority of liver genes. The TMM factor is robust for lower coverage data where more genes with zero counts may be expected (Figure S1a in Additional file 1) and is stable for reasonable values of the trim parameters (Figure S1b in Additional file 1). Using TMM normalization in a statistical test for DE (see Materials and methods) results in a similar number of genes significantly higher in liver (47%) and kidney (53%). By contrast, the standard normalization (to the total number of reads as originally used in [6]) results in the majority of DE genes being significantly higher in kidney (77%). Notably, less than 70% of the genes identified as DE using standard normalization are still detected after TMM normalization (Table 1). In addition, we find the log-fold-changes for a large set of housekeeping genes (from [16]) are, on average, offset from zero very close to the estimated TMM factor, thus giving credibility to our robust estimation procedure. Furthermore, using the non-adjusted testing procedure, 8% and 70% of the housekeeping genes are significantly up-regulated in liver and kidney, respectively. After TMM adjustment, the proportion of DE housekeeping genes changes to 26% and 41%, respectively, which is a lower total number and more symmetric between the two tissues. Of course, the bias in log-ratios observed in RNA-seq data is not observed in microarray data (from the same sources of RNA), assuming the microarray data have been appropriately normalized (Figure S2 in Additional file 1). Taken together, these results indicate a critical role for the normalization of RNA-seq data.

**Table 1:** Number of genes called differentially expressed between liver and kidney at a false discovery rate <0.001 using different normalization methods

	Library size normalization	TMM normalization	Overlap
Higher in liver	2,355	4,293	2,355
Higher in kidney	8,332	4,935	4,935
Total	10,867	9,228	7,290
House keeping genes (545)			
Higher in liver	45	137	45
Higher in kidney	376	220	220
Total	421	357	265

TMM, trimmed mean of M values.

### Other datasets

The global shift in log-fold-change caused by RNA composition differences occurs at varying degrees in other RNA-seq datasets. For example, an M versus A plot for the Cloonan *et al*. [12] dataset (Figure S3 in Additional file 1) gives an estimated TMM scaling factor of 1.04 between the two samples (embryoid bodies versus embryonic stem cells), sequenced on the SOLiD™ system. The M versus A plot for this dataset also highlights an interesting set of genes that have lower overall expression, but higher in embryoid bodies. This explains the positive shift in log-fold-changes for the remaining genes. The TMM scale factor appears close to the median log-fold-changes amongst a set of approximately 500 mouse housekeeping genes (from [17]). As another example, the Li *et al*. [18] dataset, using the llumina 1G Genome Analyzer, exhibits a shift in the overall distribution of log-fold-changes and gives a TMM scaling factor of 0.904 (Figure S4 in Additional file 1). However, there are sequencing-based datasets that have quite similar RNA outputs and may not need a significant adjustment. For example, the small-RNA-seq data from Kuchenbauer *et al*. [19] exhibits only a modest bias in the log-fold-changes (Figure S5 in Additional file 1).

Spike-in controls have the potential to be used for normalization. In this scenario, small but known amounts of RNA from a foreign organism are added to each sample at a specified concentration. In order to use spike-in controls for normalization, the ratio of the concentration of the spike to the sample must be kept constant throughout the experiment. In practice, this is difficult to achieve and small variations will lead to biased estimation of the normalization factor. For example, using the spiked-in DNA from the Mortazavi *et al*. data set [11] would lead to unrealistic normalization factor estimates (Figure S6 in Additional file 1). As with microarrays, it is generally more robust to carefully estimate normalization factors using the experimental data (for example, [20]).

### Simulation studies

**Figure 2:**
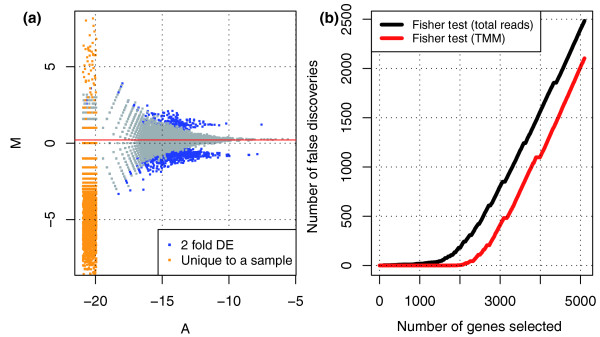
**Simulations show TMM normalization is robust and outperforms library size normalization**. **(a) **An example of the simulation results showing the need for normalization due to genes expressed uniquely in one sample (orange dots) and asymmetric DE (blue dots). **(b) **A lower false positive rate is observed using TMM normalization compared with standard normalization.

To investigate the range in utility of the TMM normalization method, we developed a simulation framework to study the effects of RNA composition on DE analysis of RNA-seq data. To start, we simulate data from just two libraries. We include parameters for the number of genes expressed uniquely to each sample, and parameters for the proportion, magnitude and direction of differentially expressed genes between samples (see Material and methods). Figure 2a shows an M versus A plot for a typical simulation including unique genes and DE genes. By simulating different total RNA outputs, the majority of non-DE genes have log-fold-changes that are offset from zero. In this case, using TMM normalization to account for the underlying RNA composition leads to a lower number of false detections using a Fisher's exact test (Figure 2b). Repeating the simulation a large number of times across a wide range of simulation parameters, we find good agreement when comparing the true normalization factors from the simulation with those estimated using TMM normalization (Figure S7 in Additional file 1).

To further compare the performance of the TMM normalization with previously used methods in the context of the DE analysis of RNA-seq data, we extend the above simulation to include replicate sequencing runs. Specifically, we compare three published methods: length-normalized count data that have been log transformed and quantile normalized, as implemented by Cloonan *et al*. [12], a Poisson regression [6] with library size and TMM normalization and a Poisson exact test [8] with library size and TMM normalization. We do not compare directly with the normalization proposed in Balwierz *et al*. [13] since the liver and kidney dataset do not appear to follow a power law distribution and have quite distinct count distributions (Figure S8 in Additional file 1). Furthermore, in light of the RNA composition bias we observe, it is not clear whether equating the count distributions across samples is the most logical procedure. In addition, we do not directly compare the normalization to virtual length [2] or RPKM [11] normalization, since a statistical analysis of the transformed data was not mentioned. However, we illustrate with M versus A plots that their normalization does not completely remove RNA composition bias (Figures S9 and S10 in Additional file 1).

**Figure 3:**
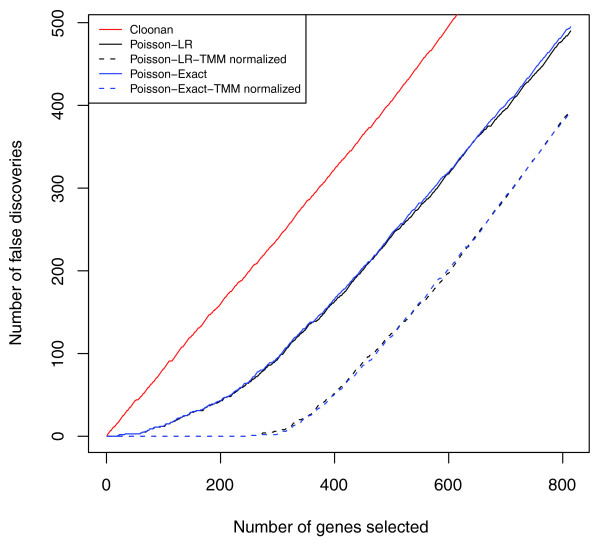
**False discovery plots comparing several published methods**. The red line depicts the length-normalized moderated t-statistic analysis. The solid and dashed lines show the library size normalized and TMM normalized Poisson model analysis, respectively. The blue and black lines represent the LR test and exact test, respectively. It can be seen that the use of TMM normalization results in a much lower false discovery rate.

For the simulation, we used an empirical joint distribution of gene lengths and counts, since the Cloonan *et al*. procedure requires both. We made the simulation data Poisson-distributed to mimic technical replicates (Figure S11 in Additional file 1). Figure 3a shows false discovery plots amongst the genes that are common to both conditions, where we have introduced 10% unique-to-group expression for the first condition, 5% DE at a 2-fold level, 80% of which is higher in the first condition. The approach that uses methodology developed for microarray data performs uniformly worse, as one might expect since the distributional assumptions for these methods are quite different. Among the remaining methods (Poisson likelihood ratio statistic, Poisson exact statistic), performance is very similar; again, the TMM normalization makes a dramatic improvement to both.

## Conclusions

TMM normalization is a simple and effective method for estimating relative RNA production levels from RNA-seq data. The TMM method estimates scale factors between samples that can be incorporated into currently used statistical methods for DE analysis. We have shown that normalization is required in situations where the underlying distribution of expressed transcripts between samples is markedly different. The assumptions behind the TMM method are similar to the assumptions commonly made in microarray normalization procedures such as lowess normalization [21] and quantile normalization [22]. Therefore, adequately normalized array data do not show the effects of different total RNA output between samples. In essence, both microarray and TMM normalization assume that the majority of genes, common to both samples, are not differentially expressed. Our simulation studies indicate that the TMM method is robust against deviations to this assumption up to about 30% of DE in one direction. For many applications, this assumption will not be violated.

One notable difference with TMM normalization for RNA-seq is that the data themselves do not need to be modified, unlike microarray normalization and some implemented RNA-seq strategies [11,12]. Here, the estimated normalization factors are used directly in the statistical model used to test for DE, while preserving the sampling properties of the data. Because the data themselves are not modified, it can be used in further applications such as comparing expression between genes.

Normalization will be crucial in many other applications of high throughput sequencing where the DNA or RNA populations being compared differ in their composition. For example, chromatin immunoprecipitation (ChIP) followed by next generation sequencing (ChIP-seq) may require a similar adjustment to compare between samples containing different repertoires of bound targets. Interestingly, the PeakSeq method [23] uses a linear regression on binned counts across the genome to estimate a scaling factor between two ChIP populations to account for the different coverages. This is similar in principle to what is proposed here, but possibly less robust. We demonstrated that there are numerous biological situations where a composition adjustment will be required. In addition, technical artifacts that are not fully captured by the library size adjustment can be accounted for with the empirical adjustment. Furthermore, it is not clear that DNA spiked-in at known concentrations will allow robust estimation of normalization factors.

Similar to previous high throughput technologies such as microarrays, normalization is an essential step for inferring true differences in expression between samples. The number of reads for a gene is dependent not only on the gene's expression level and length, but also on the population of RNA from which it originates. We present a straightforward and effective empirical method for normalization of RNA-seq data.

## Materials and methods

### TMM normalization details

A trimmed mean is the average after removing the upper and lower x% of the data. The TMM procedure is doubly trimmed, by log-fold-changes  (sample *k *relative to sample *r *for gene *g*) and by absolute intensity (*A*_*g*_). By default, we trim the *M*_*g *_values by 30% and the *A*_*g *_values by 5%, but these settings can be tailored to a given experiment. The software also allows the user to set a lower bound on the A value, for instances such as the Cloonan *et al*. dataset (Figure S1 in Additional file 1). After trimming, we take a weighted mean of *M*_*g*_, with weights as the inverse of the approximate asymptotic variances (calculated using the delta method [24]). Specifically, the normalization factor for sample *k *using reference sample *r *is calculated as:

The cases where *Y*_*gk *_= 0 or *Y*_*gr *_= 0 are trimmed in advance of this calculation since log-fold-changes cannot be calculated; G* represents the set of genes with valid *M*_*g *_and *A*_*g *_values and not trimmed, using the percentages above. It should be clear that .

As Figure 2a indicates, the variances of the M values at higher total count are lower. Within a library, the vector of counts is multinomial distributed and any individual gene is binomial distributed with a given library size and proportion. Using the delta method, one can calculate an approximate variance for the *M*_*g*_, as is commonly done with log relative risk, and the inverse of these is used to weight the average.

We compared the weighted with the unweighted trimmed mean as well as an alternative robust estimator (robust linear model) over a range of simulation parameters, as shown in Figure S4 in Additional file 1.

### Housekeeping genes

Human housekeeping genes, as described in [16], were downloaded from [25] and matched to the Ensembl gene identifiers using the Bioconductor [26] biomaRt package [27]. Similarly, mouse housekeeping genes were taken to be the approximately 500 genes with lowest coefficient of variation, as calculated by de Jonge *et al*. [17].

### Statistical testing

For a two-library comparison, we use the sage.test function from the CRAN statmod package [28] to calculate a Fisher exact *P*-value for each gene. To apply TMM normalization, we replace the original library sizes with 'effective' library sizes. For two libraries, the effective library sizes are calculated by multiplying/dividing the square root of the estimated normalization factor with the original library size.

For comparisons with technical replicates, we followed the analysis procedure used in the Marioni *et al*. study [6]. Briefly, it is assumed that the counts mapping to a gene are Poisson-distributed, according to:

where  represents the fraction of total reads for gene *g *in experimental condition *z*_*k*_. Their analysis utilizes an offset to account for the library size and a likelihood ratio (LR) statistic to test for differences in expression between libraries (that is, H_0_:*μ*_*g*1 _= *μ*_*g*2_). In order to use TMM normalization, we augment the original offset with the estimated normalization factor. The same LR testing framework is then used to calculate *P*-values for DE between tissues. We modified this analysis to use an exact Poisson test for testing the difference between two replicated groups. The strategy is similar in principle to the Fisher's exact test: conditioning on the total count, we calculated the probability of observing group counts as or more extreme than what we actually observed. The total and group total counts are all Poisson distributed.

We re-implemented the method from Cloonan *et al*. [12] for the analysis of simulated data using a custom R [29] script.

### Simulation details

The simulation is set up to sample a dataset from a given empirical distribution of read counts (that is, from a distribution of observed *Y*_*g*_). The mean is calculated from the sampled read counts divided by the sum *S*_*k *_and multiplied by a specified library size *N*_*k *_(according to the model). The simulated data are then randomly sampled from a Poisson distribution, given the mean. We have parameters specifying the number of genes common to both libraries and the number of genes unique to each sample. Additional parameters specify the amount, direction and magnitude of DE as well as the depth of sequencing (that is, range of total numbers of reads). Since we have inserted known differentially expressed genes, we can rank genes according to various statistics and plot the number of false discoveries as a function of the ranking. Table S1 in Additional file 1 gives the parameter settings used for the simulations presented in Figures 2 and 3.

### Software

Software implementing our method was released within the edgeR package [30] in version 2.5 of Bioconductor [26] and is available from [31]. Scripts and data for our analyses, including the simulation framework, have been made available from [32].

## Abbreviations

ChIP: chromatin immunoprecipation; DE: differential expression; LR: likelihood ratio; RPKM: reads per kilobase per million mapped; TMM: trimmed mean of M values.

## Authors' contributions

MDR and AO conceived of the idea, analyzed the data and wrote the paper.

## Supplementary Material

Additional file 1 A Word document with supplementary materials, including 11 supplementary figures and one supplementary table. Click here for file
