# Supplementary material for: A scaling normalization method for differential expression analysis of RNA-seq data
Source: Genome Biol. 2010 Mar 2;11(3):R25. doi: 10.1186/gb-2010-11-3-r25 (PMC2864565; doi:10.1186/gb-2010-11-3-r25)
Supplement: Additional file 1 — A Word document with supplementary materials, including 11 supplementary figures and one supplementary table. [file gb-2010-11-3-r25-S1.doc]

Material for: **A scaling normalization method for differential expression analysis of RNA-seq data**. Mark D. Robinson and Alicia Oshlack.


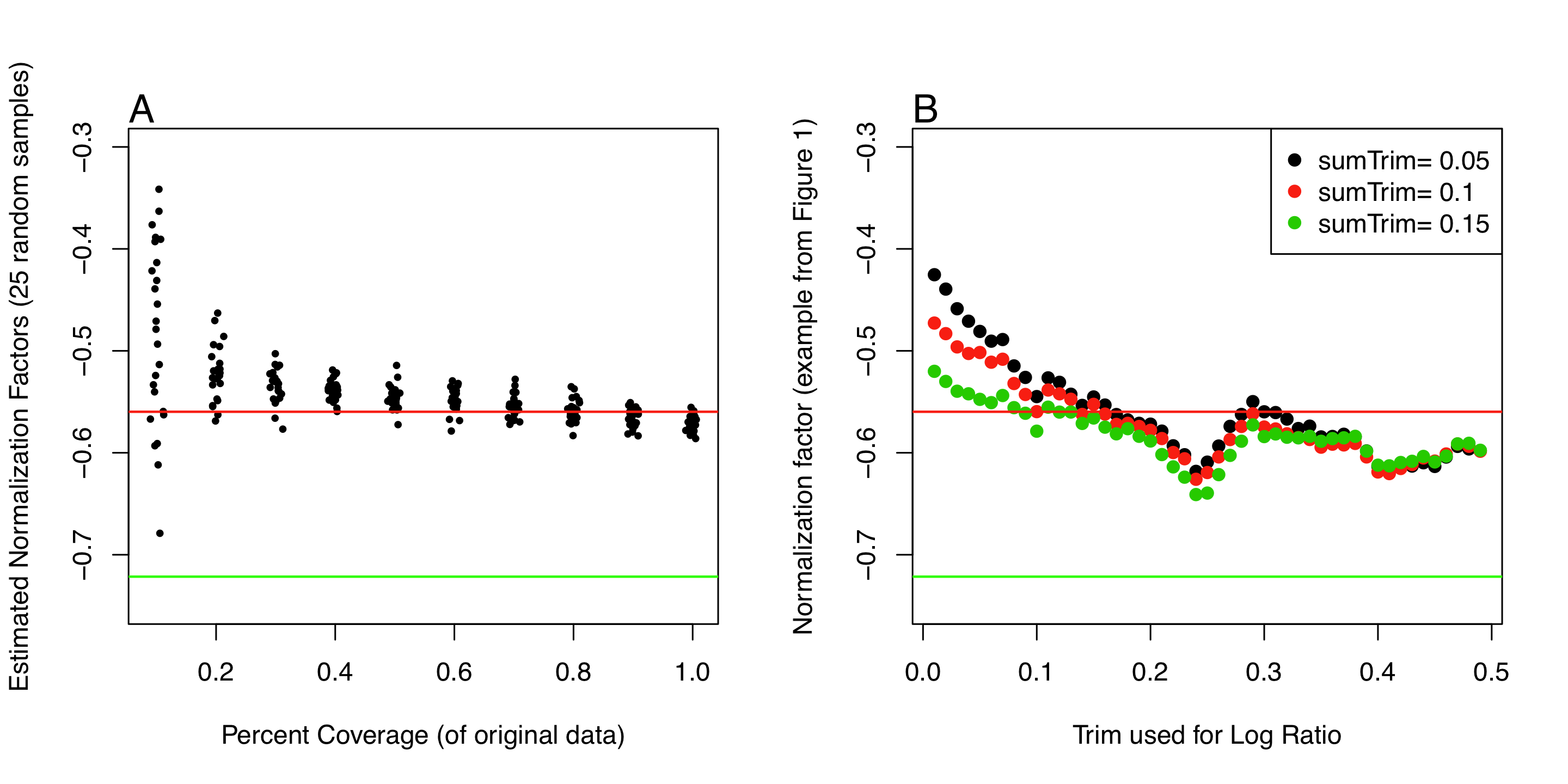


**Figure S1**. A. Estimated normalization factors for 2 samples from the Marioni et al. dataset (from Figure 1) using 25 random subsamples of the original data at 10 levels of coverage. B. Estimated normalization factors using various settings of log-ratio trim (X-axis) and trim on the A values (sumTrim dot colour). On both panels, the red line represents the estimated normalization factor and the green line represents the median log-ratio of the housekeeping genes. Note that the scale of the Y-axis in these plots is within a small range compared to the entire data range.


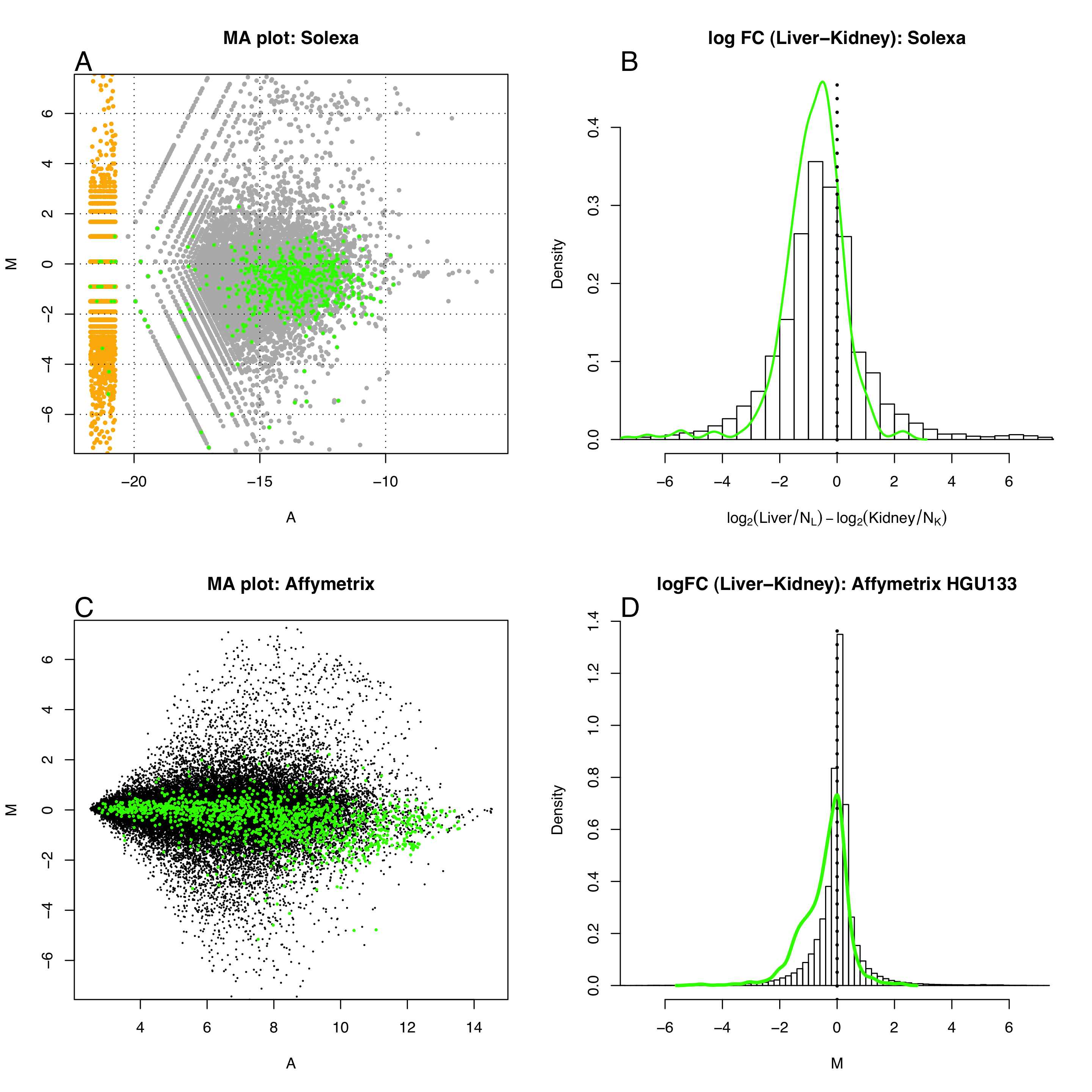


**Figure S2**. A,B. M-versus-A plot and histogram of log-ratios between liver and kidney from the Marioni et al. RNA-seq data, recreated from Figure 1C and 1B, respectively, for easy comparison. C. Corresponding M-versus-A plot for the liver versus kidney comparison from Affymetrix microarray data (using RNA from the same source). D. Corresponding histogram of log-ratios from the liver versus kidney comparison using the Affymetrix microarray data. The green dots/lines represent the housekeeping genes. Note that there is **no** major shift in the entire distribution of log-ratios for the microarray data. In addition, the mode of the housekeeping genes log-ratio is at 0 for the microarray data.


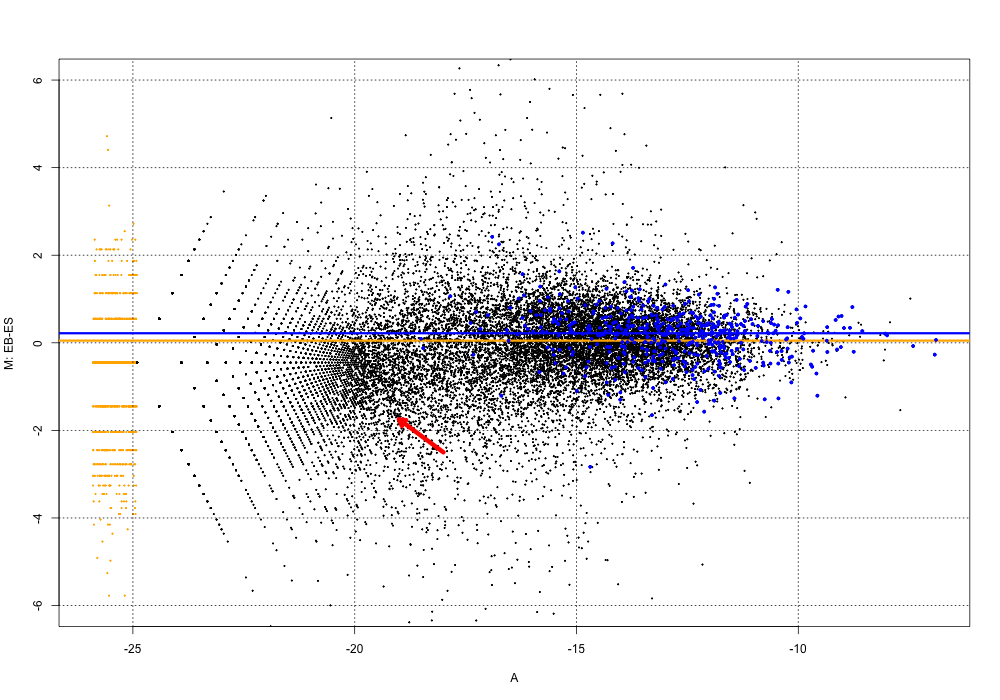


**Figure S3**. M-versus-A plots for the RNA-seq dataset comparing mouse embryoid bodies and embryonic stem cells (Cloonan et al. 2008). Approximately 500 “housekeeping” genes (using summaries from de Jonge et al. 2007) are highlighted in blue. The blue line represents the median log-fold-change amongst the housekeeping genes. The orange line indicates the estimated TMM scale factor. The red arrow highlights an interesting set of genes that are offset in the negative direction; this contributes to the positive shift observed for the housekeeping and remainder of the genes. We used an A cutoff of -16 for the TMM estimate in order remove the effect of these genes.


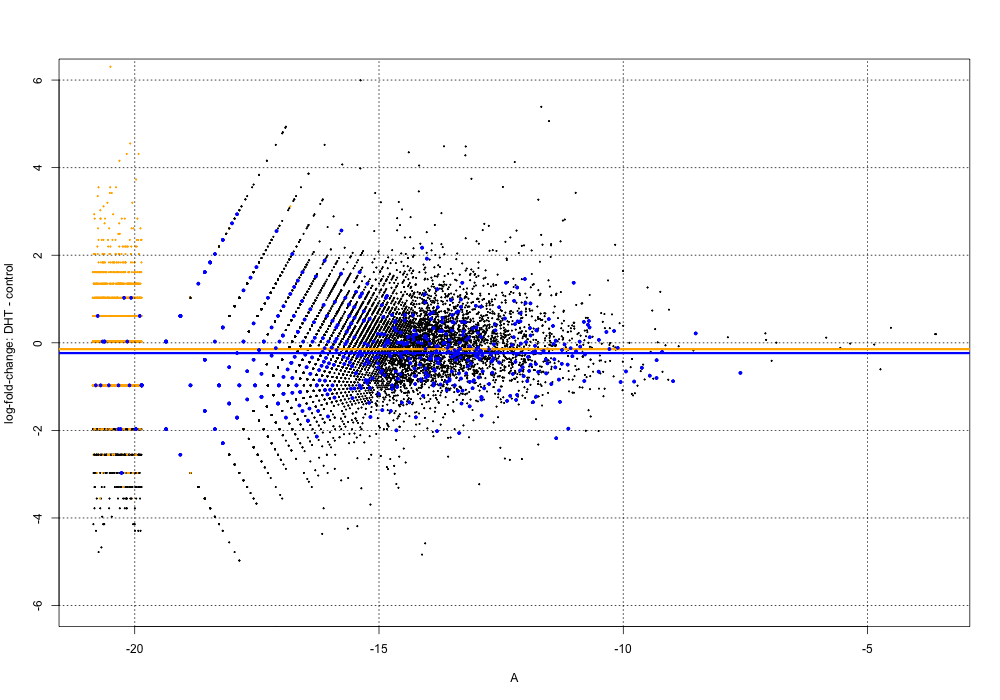


**Figure S4**. M-versus-A plots for the RNA-seq dataset comparing DHT-stimulated versus unstimulated LNCaP cells. (Li et al. 2008). Approximately 500 “housekeeping” genes (from Eisenberg and Levanon, 2003) are highlighted in blue. The blue line represents the median log-fold-change amongst the housekeeping genes. The orange line indicates the estimated TMM scale factor.


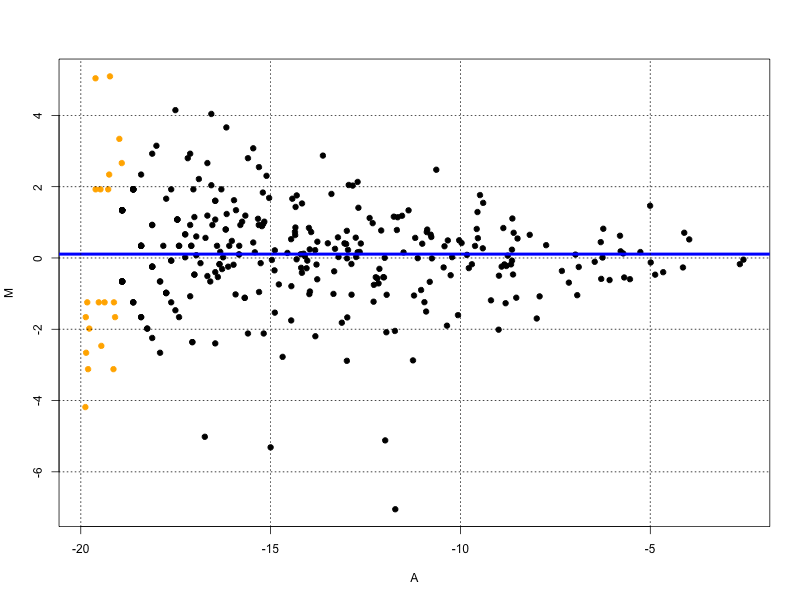


**Figure S5**. M-versus-A plots for the microRNA data comparing a preleukemic (ND13) and leukemic cell line (ND13+Meis1) (Kuchenbauer et al., 2008). Here, we see a slight positive offset of the M-values (blue line), due largely to the handful of small RNA sequences that are strongly expressed in ND13 cells.


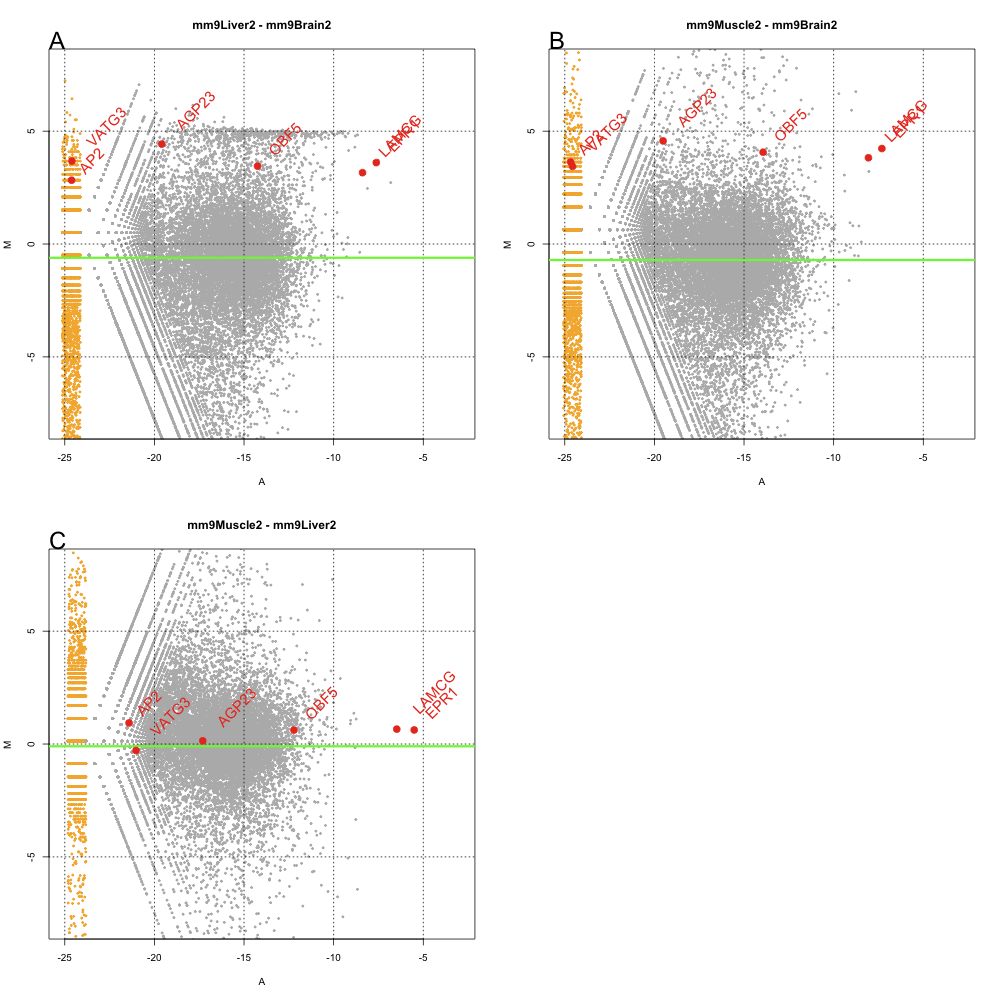


**Figure S6**. M-versus-A plots for the 3 possible comparisons between Liver, Brain and Muscle from the Mortazavi et al. dataset. Each dot represents a gene (a table of gene counts was created from the unique reads mapping to the genome only). The red dots represents the non-mouse spiked-in genes. The green lines represent the TMM normalization factors.


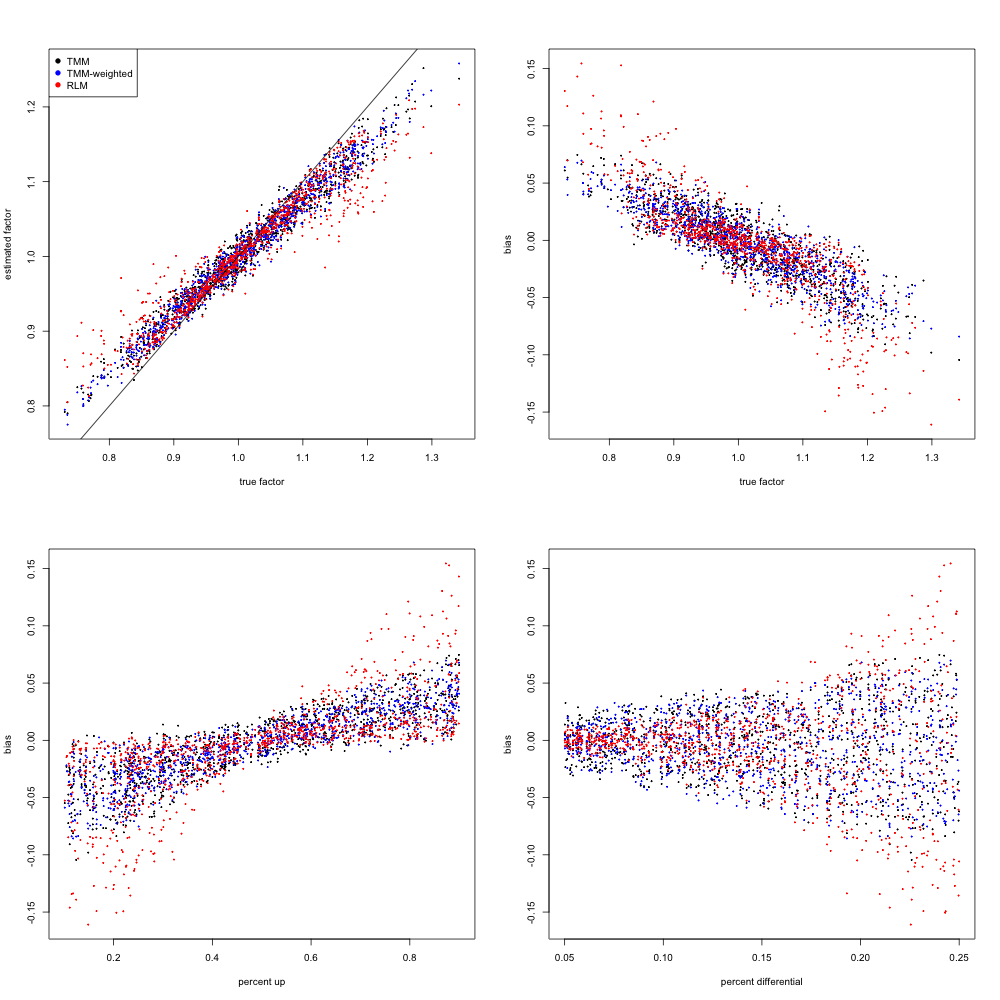


**Figure S7**. Exploratory analysis of the scaling factors estimation procedure, across a broad range of simulation parameters for 2 simulated samples (20000 genes, proportion upregulated ~ Uniform(.1,.9), proportion differential ~ Uniform(.05,.25), # genes unique to group ~ Uniform(0,2000), 4-fold differential expression). In all plots, the black points represent the estimates with the unweighted trimmed mean (trim=.45), weighted trimmed mean (logratiotrim=.25, Avaluetrim=.05) and a robust linear model with MM estimation. The top left panel plots the estimated factors versus the true factors; the general agreement is quite good and log binomial weighting does provide an improvement. The top right panel shows the bias versus the true factor. The bottom left panel shows the bias as a function of the degree of asymmetry in the differential expression; the bias increases with asymmetry and the log binomial weighting results in less bias. The bottom right panel shows the bias as a function of the percentage of differential genes; here, the variability of the bias increases with the percentage of differentially expressed genes but to a lesser degree for the weighted trimmed mean.


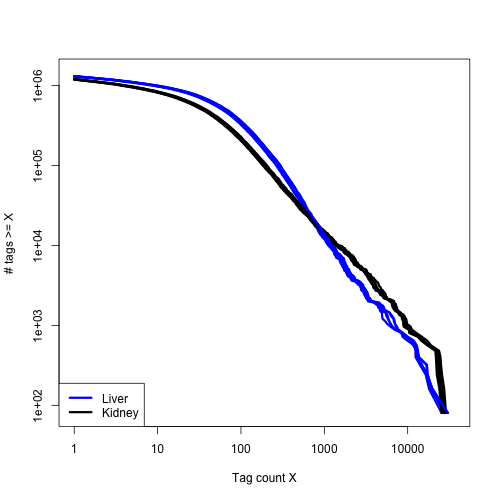


**Figure S8**. Reverse cumulative distribution plot, as discussed in Balwierz et al. for the Marioni et al. dataset. X-axis plots a gene count and the Y-axis gives the number of genes with a count of at least the X value. The liver and kidney distribution show distinct reverse cumulative distributions.


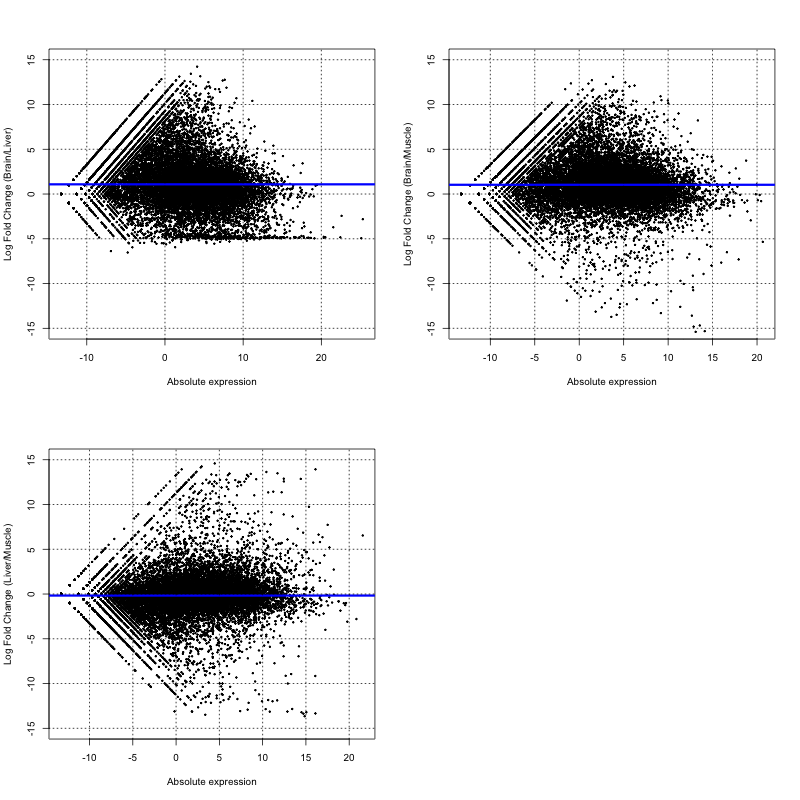


**Figure S9**. M-versus-A plots for the RPKM-normalized RNA-seq data from Mortazavi et al. 2008. The three plots give log-fold changes for the 3 pairs of mouse tissues (left to right, top to bottom: Brain to Liver, Brain to Muscle and Liver to Muscle), as indicated. Each dot represents a gene. Blue lines indicate trimmed mean log fold changes. The first two of these are offset significantly from zero, possibly due to the RPKM normalization not accounting for the composition bias.


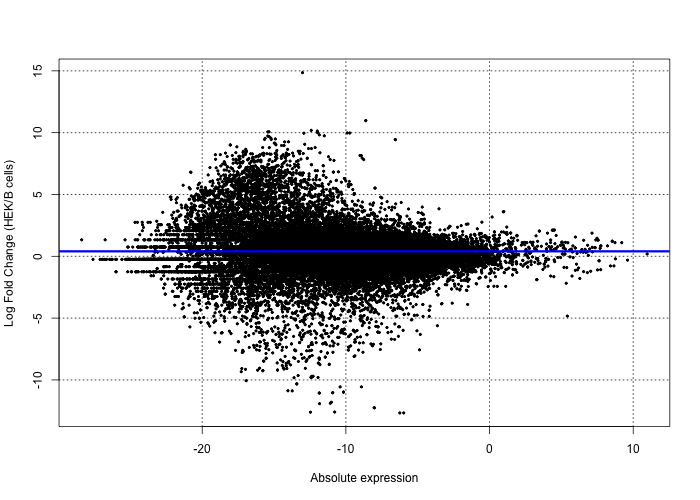


**Figure S10**. M-versus-A plots for the “virtual length”-normalized data from Sultan et al. 2008 comparing HEK and B cells. Each dot represents a gene. The blue line indicates trimmed mean log fold changes. The trimmed mean is somewhat offset from zero, possibly due to the virtual length normalization not accounting for the composition bias.


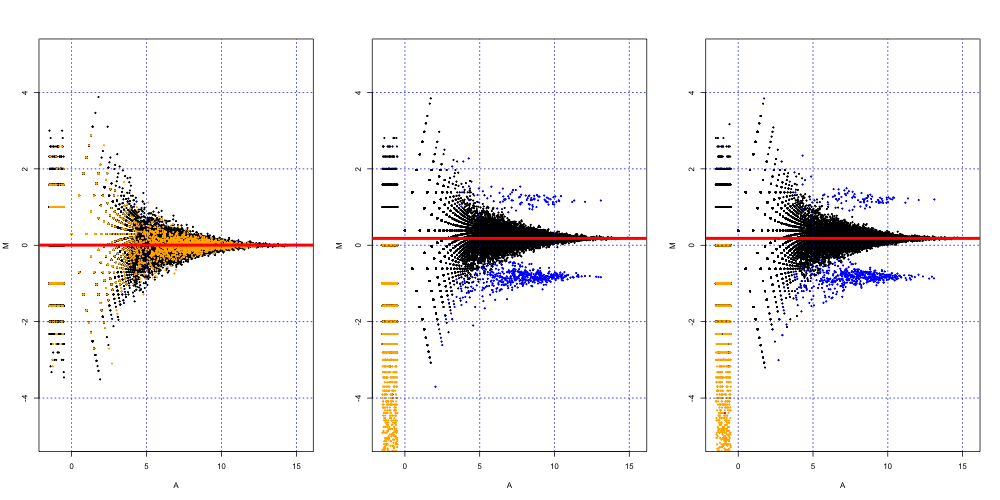


**Figure S11**. M-versus-A plots for the simulation of replicated Poisson distributed samples, relative to the reference. The left panel is the replicate of the reference, the middle and right panels are the two libraries from the other experimental condition compared to the reference. Here, the count distribution is taken from the empirical distribution of RNA-seq counts from EB cells (Cloonan et al. 2008), using the same gene length distribution. The parameter settings are 5% differentially expressed genes (blue dots) at 2-fold, 80% in 1 direction, and 10% unique-to-one-group expression (orange dots).

**Table S1**. Parameter settings for the simulations presented in Figures 2 and 3.

|  | Figure 2 | Figure 3 |
| --- | --- | --- |
| Comparison | 1 library versus 1 library | 2 libraries versus 2 libraries |
| Empirical distribution of read counts | Kidney sample from Marioni et al. | EB sample from Cloonan et al. |
| Number of common genes | 20,000 | 20,000 |
| Number of genes unique to: | Sample 1: 3000  Sample 2: 100 | Group 1: 2200  Group 2: 0 |
| Range of sampled library sizes | 1,000,000 | 600,000 – 1,000,000 |
| Percent DE (among common genes) | 10% | 5% |
| Percent “up”-regulated (among DE genes) | 80% | 80% |
| Fold change of DE genes | 2 | 2 |
